# Amorphous Solid Forms of Ranolazine and Tryptophan and Their Relaxation to Metastable Polymorphs

**DOI:** 10.1021/acs.cgd.3c00565

**Published:** 2023-08-18

**Authors:** Joana F. C. Silva, Pedro S. Pereira Silva, Manuela Ramos Silva, Elvira Fantechi, Laura Chelazzi, Samuele Ciattini, M. Ermelinda S. Eusébio, Mário T. S. Rosado

**Affiliations:** † CQC-IMS, Departamento de Química, Faculdade de Ciências e Tecnologia, Universidade de Coimbra, 3004-535 Coimbra, Portugal; ‡ CFisUC, Departamento de Física, Faculdade de Ciências e Tecnologia, Universidade de Coimbra, Rua Larga, 3000-370 Coimbra, Portugal; § Centro di Cristallografia Strutturale (CRIST), Università degli Studi di Firenze, Via della Lastruccia 3, Sesto Fiorentino 50019 Firenze, Italy

## Abstract

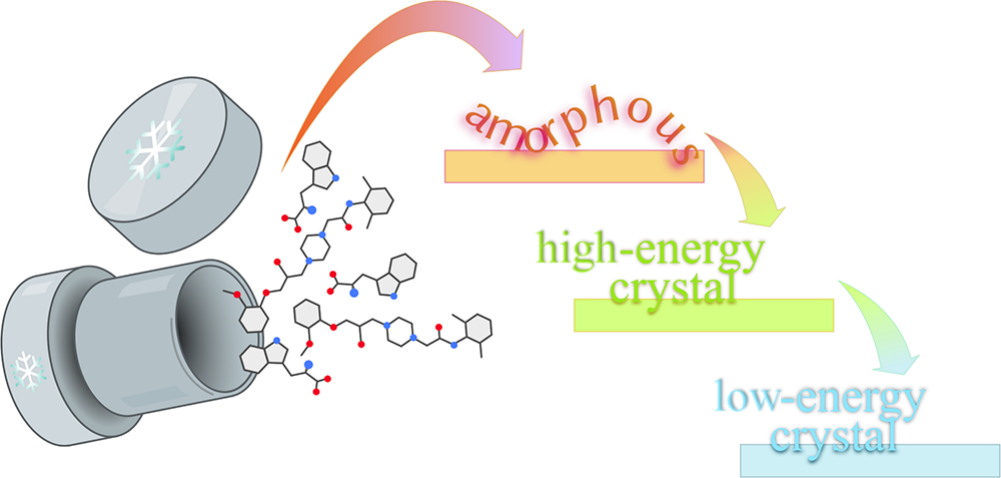

Different methods were explored for the amorphization of ranolazine, a sparingly soluble anti-anginal drug, such as mechanochemistry, quench-cooling, and solvent evaporation from solutions. Amorphous phases, with *T*_g_ values lower than room temperature, were obtained by cryo-milling and quench-cooling. New forms of ranolazine, named II and III, were identified from the relaxation of the ranolazine amorphous phase produced by cryo-milling, which takes place within several hours after grinding. At room temperature, these metastable polymorphs relax to the lower energy polymorph I, whose crystal structure was solved in this work for the first time. A binary co-amorphous mixture of ranolazine and tryptophan was produced, with three important advantages: higher glass transition temperature, increased kinetic stability preventing relaxation of the amorphous to crystalline phases for at least two months, and improved aqueous solubility. Concomitantly, the thermal behavior of amorphous tryptophan obtained by cryo-milling was studied by DSC. Depending on experimental conditions, it was possible to observe relaxation directly to the lower energy form or by an intermediate metastable crystalline phase and the serendipitous production of the neutral form of this amino acid in the pure solid phase.

## Abstract 2

Amorphous phases of ranolazine, tryptophan, and co-amorphous mixtures were produced and their stepwise relaxations toward low-energy crystals were investigated. Unprecedently, three polymorphs of ranolazine were discovered and the crystal structure of the most stable solved. Amorphous tryptophan relaxation led to the serendipitous appearance of its neutral form. A co-amorphous mixture showed enhanced thermal and kinetical stability and improved ranolazine aqueous solubility.

## 1 Introduction

Amorphous phases lack long-range structural order, although they may present some short-range order.^1^ An amorphous solid may be regarded as structurally similar to a super-cooled liquid, with very high viscosity. They are kinetically restricted, trapped in one among numerous shallow minima in the potential energy landscape, a high-energy state locally surrounded by relatively low potential energy barriers.^2,3^ These metastable disordered phases are prone to spontaneously relax to a lower energy state, i.e., crystalline solid.^4,5^ Several factors, thermodynamic and kinetic, can determine recrystallization outcome from an amorphous state.^6^ Coming from a much higher energy state, relaxation of an amorphous phase can lead to a metastable crystal, with the inherent random orientations in disordered phases leading to intermolecular interactions different of those present in the most stable crystal.^5^ Notwithstanding, these reaction paths following the Ostwald step rule are frequent but not always followed.^7^

Most active pharmaceutical ingredients (APIs) are administered orally in crystalline solid phases with low aqueous solubility. However, low solubility and dissolution rate are detrimental to drug bioavailability and compromise the development of oral pharmaceutical forms.^8^ Amorphization has been recognized as one of the most promising approaches to improve the oral bioavailability of APIs. Therefore, the investigation of the relaxation of amorphous phases to crystalline forms is highly relevant for the development of solid drug formulations based on these metastable materials. Additionally, co-amorphization with small molecules is an emergent approach to prevent relaxation to crystalline drugs, kinetically stabilizing the desired disordered solid phase by intimate interaction with low-molecular-weight excipients and/or other drug molecules. Co-amorphous systems can be stabilized by different kinds of interactions between the drug and the co-former or by the effect of segregation in mixing.^9,10^

**Figure 1:**
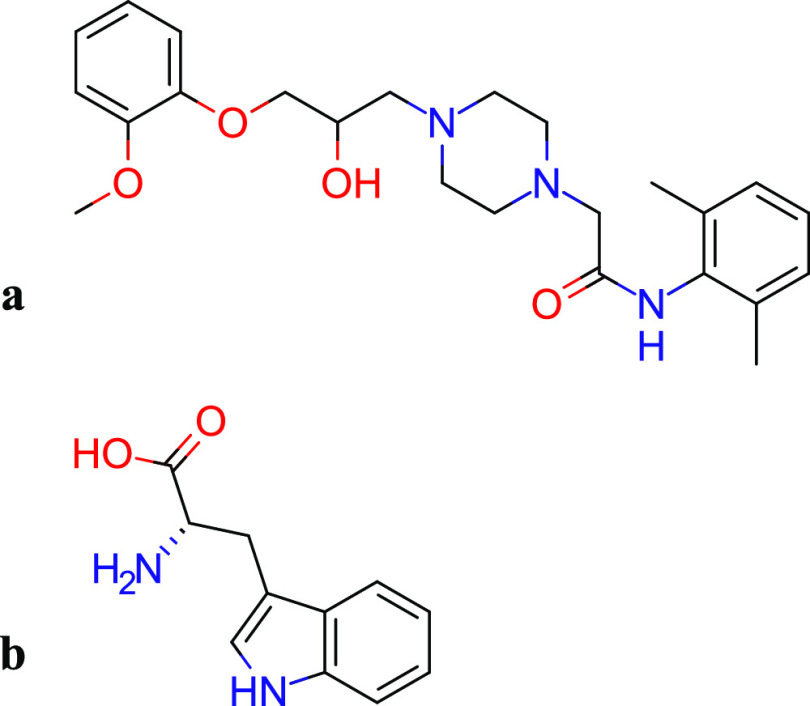
Molecular structure^19^ of (a) ranolazine and (b) l-tryptophan.

Ranolazine (RNL), Figure 1a, is an anti-anginal agent widely used in treating cardiovascular diseases, including arrhythmias, variant and exercise-induced angina, and myocardial infarction. Ranolazine shows advantages over other anti-anginal agents because it exhibits an anti-ischemic effect, which is not influenced by either blood pressure or heart rate.^11,12^ Bioavailability of RNL is limited by its low aqueous solubility.^13^ The improvement of the physicochemical properties of an API depends also on the co-former when it is part of a multicomponent system. Amino acids are one of the most used types of co-formers in co-amorphous systems, as they are generally recognized as safe (GRAS).^14^l-Tryptophan (TRP), Figure 1b, is an essential amino acid for humans and acts as a biochemical precursor to produce the neurotransmitter serotonin and the vitamin niacin. TRP has been used recently in several co-amorphous systems.^15−18^

In this work, different methodologies, such as mechanochemistry, quench cooling, and solvent evaporation from solutions, were explored for RNL amorphization. The relaxation of the amorphous phase obtained by cryo-milling was investigated and two new metastable crystalline forms were identified. These polymorphs are monotropically related to the more stable form that they subsequently relax into, whose structure was solved for the first time. Thus, this work is also a contribution to the knowledge of the largely unexplored landscape of RNL solid forms. It also provides an opportunity to investigate the crystallization of amorphous TRP and the thermal behavior of its new metastable polymorph, whose structure was recently solved by XRPD.^20^ Finally, co-amorphization of RNL-TRP was assessed as a viable strategy to slow down relaxation to crystalline counterparts while increasing RNL aqueous solubility.

## 2 Materials and Methods

### 2.1 Materials

Ranolazine (racemic) was acquired from BLD Pharm, *x* = 0.99. Tryptophan, *x* = 0.99, and poly(ethylene oxide) (PEO) with *M*_W_ ≈ 600,000 were purchased from Sigma-Aldrich and acetonitrile, *x* = 0.998, from Fisher.

Ranolazine crystals were obtained by crystallization in a gel produced with PEO-acetonitrile, mixing 20 mg of RNL, 2 mL of acetonitrile, and 0.08 g L^–1^ of PEO according to the crystallization technique described by Choquesillo-Lazarte and García-Ruiz.^21^ Polymorph screening was performed by (1) slow evaporation recrystallization at room temperature using different analytical grade solvents, (2) rapid evaporation under vacuum at 60 °C from dichloromethane solutions, (3) heating/cooling cycles by DSC on the pure compound with different rates: |β| = 2, 10, and 20 °C min^–1^.

### 2.2 Amorphization

Amorphous materials were prepared by cryo-milling about 100 mg of powders consisting of pure RNL, pure TRP, and RNL-TRP mixtures with 2:1, 1:1, and 1:2 molar ratios. The milling process was performed with a Retsch MM400 oscillatory ball mill at a 30 Hz frequency in stainless steel 10 mL jars containing 2 balls (⌀ = 7 mm). During each 60 min milling process, the jars were dipped in liquid nitrogen 4 times, to reduce the risk of recrystallization caused by friction heating. Each immersion lasted about two minutes, until boiling nitrogen settled.

### 2.3 Single-Crystal X-ray Diffraction (SCXRD)

Single-crystal X-ray diffraction data of racemic ranolazine were collected at 100 K using a Bruker D8 Venture diffractometer equipped with a PHOTON II detector and a microfocus source (Cu-Kα radiation, λ = 1.54184 Å). A total of 5704 frames were collected with the Bruker APEX4 program suite^22^ and integrated and reduced with the Bruker SAINT software.^23^ Data were corrected for absorption effects using the Multi-scan method (SADABS).^24^

The crystal structure was solved and refined using the Bruker SHELXTL software package, using the space group *P*2_1_/*n*, with *Z* = 4. All non-hydrogen atoms were refined with anisotropic displacement parameters, while the hydrogen atoms were set in calculated positions.

Molecular plots of crystalline structures were produced using the Mercury (2021.2.0) sofware.^25^ Crystallographic data and refinement parameters are reported in Table 1. SCXRD data have been deposited in the CSD with the deposition number 2260934.

**Table 1:** Single-Crystal X-ray Structure Determination Parameters of RNL

name	ranolazine
formula	C_24_H_33_N_3_O_4_
*M*_W_/g mol^–1^	427.53
*T*/K	100(2)
λ/Å	1.54178
crystal system, space group	monoclinic, *P*2_1_/*n*
unit cell dimensions/(Å, °)	*a* = 8.6212(7)
	*b* = 7.1560(6)
	*c* = 35.741(3)
	β = 95.078(4)
volume/Å^3^	2196.3(3)
*Z*, dc/g cm^–3^	4, 1.290
μ/mm^–1^	0.713
*F*(000)	916
transmission factors (min/max)	0.9580/0.9860
crystal size (mm)	0.02 × 0.05 × 0.06
reflection collected/unique (*R*_int_)	25,000/4009 (0.0968)
2θ range/°	2.48/67.679
data/parameters	4009/289
final *R* indices [*I* > 2σ]	*R*_1_ = 0.1172, w*R*_2_ = 0.3354
*R* indices (all data)	*R*_1_ = 0.1415, w*R*_2_ = 0.3986
GOF	1.074

### 2.4 X-ray Powder Diffraction (XRPD)

The X-ray powder diffraction measurements were carried out in a Bruker D8 Advance diffractometer with a Bragg–Brentano reflection geometry, using filtered Cu Kα (λ = 1.5418 Å) with nickel. The diffractograms were collected in the 2θ range of 4° to 50°, with a step of 0.02° and 0.5 s accumulation time per step. A 1D Brucker LINXEYE multidetector was used with energy discrimination for background noise reduction, using 196 pixels corresponding to an equivalent acquisition time of 0.5 × 196 = 96 s per step on a point detector. The incident beam was limited by a divergence slit of 0.3°, and Soller slits of 0.5° were also used to limit the divergence of the incident and diffracted beams in the direction perpendicular to the diffraction plane.

### 2.5 Variable Temperature X-ray Powder Diffraction (VT-XRPD)

Variable temperature X-ray powder diffraction measurements were performed with a Bruker D8 Advance diffractometer with Bragg–Brentano reflection geometry, using CuKα (λ = 1.5418 Å) radiation filtered from Kβ using nickel, a 1D Brucker LINXEYE discrimination energy multidetector, divergence slit of 0.6°, and 2.5° Soller slits. Measurements were performed under nitrogen atmosphere using a Wide Range MRI sample chamber able to run from −125 to 175 °C. The diffractograms were collected in the 2θ range of 3° to 40°, with a step of 0.03° and 0.25 s as time per step.

### 2.6 Scanning Electron Microscopy (SEM)

The microstructural analysis of the surfaces was performed using a TESCAN VEGA 3 SBH - Easy Probe SEM with a tungsten heated cathode. The SEM images were acquired with a working voltage of 5 kV and using the secondary electrons detector. The samples were coated with gold–palladium sputtering under an argon atmosphere. Quorum SC7620 Mini Sputter Coater/Glow Discharge System with a gold/palladium (Au/Pd) sputter target was used, and the samples were coated with a 10 nm thick layer.

### 2.7 Fourier Transform Infrared Spectroscopy with Attenuated Total Reflection (FTIR-ATR)

Infrared spectra of the solids were recorded at room temperature using a Thermo Nicolet IR300 spectrometer with diamond crystal ATR accessory, accumulating 64 scans at a 2 cm^–1^ resolution. Some spectral bands were assigned to vibrational modes with the aid of calculated frequencies (B3LYP/def2-SVP) and comparison with the literature cited herein.

### 2.8 Differential Scanning Calorimetry (DSC)

Differential scanning calorimetry measurements were carried out using a Perkin-Elmer DSC7 calorimeter with an intracooler cooling unit at −20 °C (ethylene glycol–water, 1:1, V/V, cooling mixture). Measurements were carried out at different scanning rates |β| = 2, 10, and 20 °C min^–1^ under a nitrogen gas flow of 20 mL min^–1^. Samples with approximate mass between 2 and 5 mg were placed in 10 or 50 μL aluminum pans, with similar empty ones used as reference. Temperature calibration was performed with high grade standards:^26,27^ biphenyl (CRM LGC 2610, *T*_fus_ = (68.93 ± 0.03) °C) and indium (Perkin-Elmer, *x* = 0.9999, *T*_fus_ = 156.6 °C). Indium was also used for enthalpy calibration (Δ_fus_*H*_m_ = 3286 ± 13 J mol^–1^). Benzoic acid (CRM LGC 2606, *T*_fus_ = (122.35 ± 0.02) °C) was used to check the calibration. Pyris software version 3.50 was used for instrument control and result analysis. The reported first-order phase transition temperatures correspond to the onset of the peaks. The glass transition temperatures were determined from the midpoints of the characteristic baseline step changes.

### 2.9 Thermogravimetric Analysis (TGA)

Thermogravimetric analysis measurements were done using simultaneous TG/DSC STA 6000 from Perkin-Elmer. For these experiments, the samples were scanned from 25 to 600 °C at a scan rate of 10 °C min^–1^, in alumina pans for a mass sample of ≈7.5 mg under a nitrogen atmosphere.

### 2.10 Apparent Solubility

The solubilities were measured at 25 °C in Milli-Q water by the shake-flask method. Glass vials containing excess solid powder (RNL, TRYP, and RNL-TRYP co-amorphous) were kept in a constant-temperature bath at 25 °C for at least 48 h under agitation. Samples were obtained by quick filtration of slurry aliquots through a 0.45 μm Millipore membrane and appropriately diluted with 0.1 M HCl aqueous solution. The concentration of RNL was then determined by an ultraviolet (UV) absorption method using a Shimadzu UV-1800 spectrophotometer at three different wavelengths (271, 277, and 288 nm) using UVProbe 2.52 software. The relation between solute concentration and the intensity of UV absorption was calibrated prior to the experiments. The calibration curves are presented in Figure S1. All solubility experiments were performed in triplicate.

## 3 Results and Discussion

### 3.1 Ranolazine

#### 3.1.1 Single-Crystal Structure of RNL (Form I)

Given the scarcity of scientific results regarding the solid-state forms of ranolazine, one of the aims of this work was to investigate its polymorphism. We confirmed that RNL single crystals are difficult to grow. After the longest and most accurate screening that could be afforded, the best crystal specimen for single-crystal data collection was selected from the many batches available. However, despite the sub-optimal quality of the collected data, it was possible to resolve the structure of form I. Several data collections were performed with other crystals and the structures obtained were equivalent to the one here presented, but the reliability factors obtained were slightly higher. There was no evidence of disorder in all those structures.

This study employed several techniques, such as milling, melt cooling, and crystallization by solvent evaporation. Single crystals of necessary quality for structural resolution by SXRD were produced by solvent evaporation from PEO-acetonitrile gel, as described in Section 2.1.

**Figure 2:**
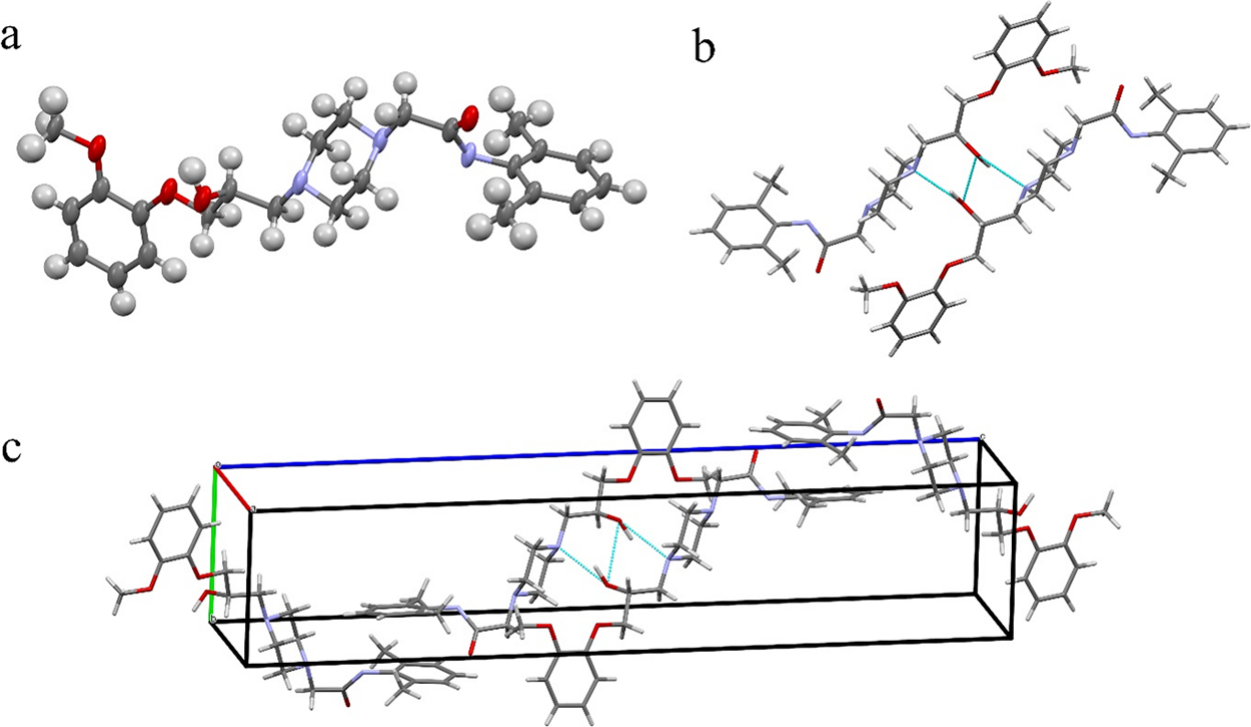
(a) Thermal ellipsoid view of the asymmetric unit of ranolazine. (b) Zoom on the central region, highlighting the dimeric stacking interactions and the hydrogen bonds N···H–O and O···H–O. (c) Unit cell contents of the ranolazine structure.

Racemic ranolazine crystallized in the monoclinic system, space group *P*2_1_/*n*, with one molecule of RNL in the asymmetric unit and four molecules of RNL in the unit cell. The representation of the asymmetric unit is reported in Figure 2a. The analysis of the packing structure, reported in Figure 2b, shows that two molecules of RNL are linked by a hydrogen bond forming a dimer at the center of the unit cell. The formation of the dimer between the two adjacent, symmetry related, molecules can occur with two different hydrogen bonds: N···H–O and O···H–O, Figure 2c. Within this framework, the hydroxyl hydrogen atom is involved in both these two hydrogen bonds. Thus, the hydroxyl hydrogen atom location is mediated between the two directions N···O and O···O. For this reason and due to the sub-optimal data quality, the coordinates were not determined from the Fourier difference density map, but they were then set in a calculated position. Finally, each dimer is related to the adjacent by stacking interactions forming a zig-zag chain along the *c* axis, Figure 2b.

**Figure 3:**
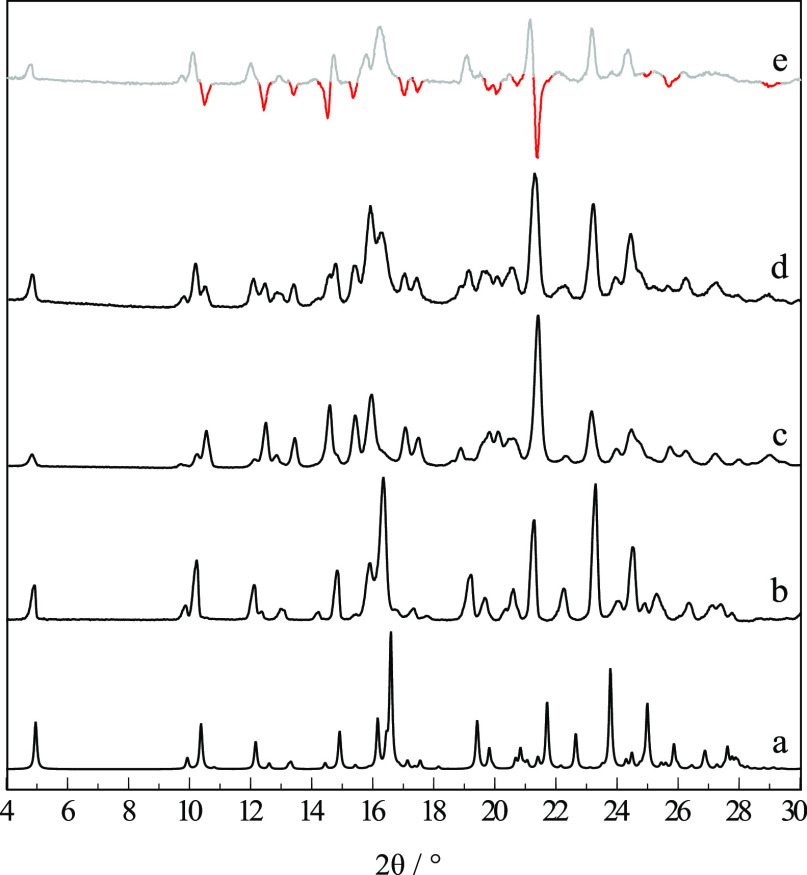
X-ray powder diffraction patterns of RNL: (a) simulated from form I crystal structure; (b) commercial starting material, form I; (c) mixture of forms I and II and/or III crystallized from the amorphous phase obtained by cryo-milling; (d) cryo-milled sample (c) kept at room temperature for a week; (e) difference between curves (d) – (c), evidencing diminishing forms III and/or II (red) and increasing form I reflections (gray).

Bulk powders of the commercial sample of RNL were analyzed by XRPD and matched the diffractogram simulated from the single crystal reported here, as shown by traces (a) and (b) in Figure 3, despite the slight 2θ shift due to different experimental temperatures. This form is hereafter identified as polymorph I.

#### 3.1.2 Physicochemical Characterization of RNL Form I

**Figure 4:**
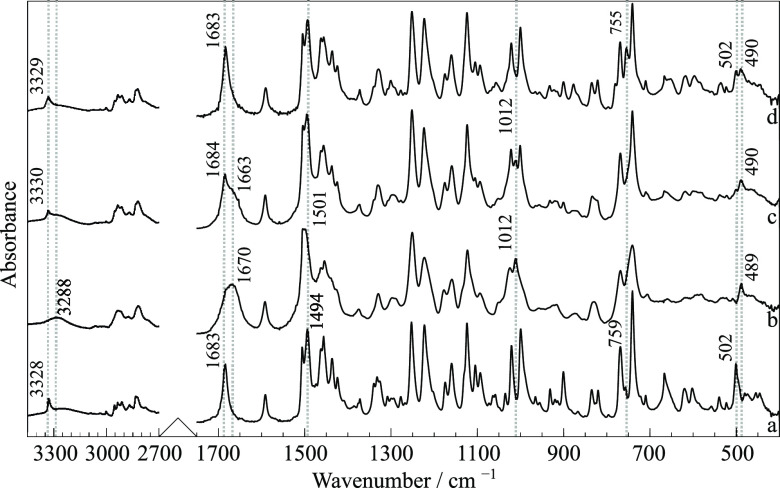
FTIR-ATR spectra of RNL. (a) Commercial sample, form I; (b) supercooled liquid RNL after cryo-milling; (c) cryo-milled sample containing a mixture of form I with form II and/or III; (d) cryo-milled sample (c) kept at room temperature for a week.

The same RNL starting material was then characterized by spectroscopic methods and thermal analysis. Despite the complexity due to the variety of functional groups in RNL, some characteristic spectral features can be assigned to vibrational modes in the FTIR-ATR spectrum, as shown in Figure 4a. Some of them are notable because of their sensitivity to intermolecular aggregation, such as those related to the amide group (ν(NH), ν(C=O), and δ(NH) at 3328, 1683, and 1494 cm^–1^, respectively). The broad band centered at 3264 cm^–1^, indicating hydrogen bonding involving the hydroxyl group, concurs with the resolved crystalline structure of polymorph I.

**Figure 5:**
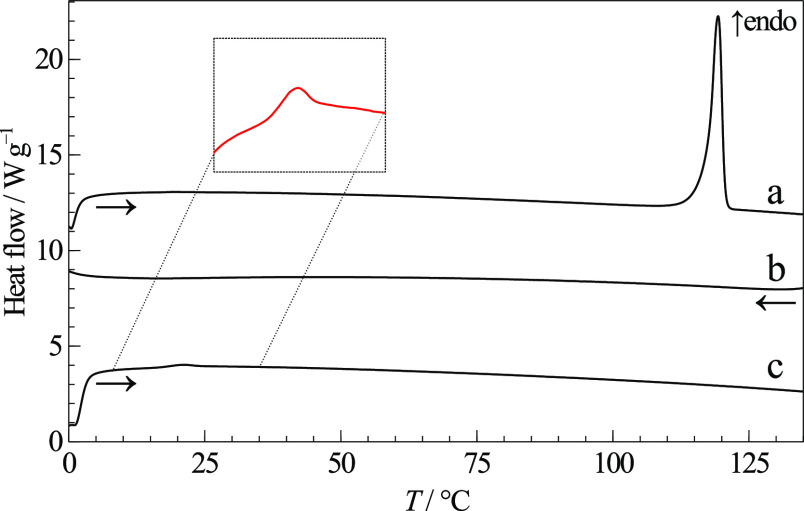
DSC thermograms showing melting and quench cooling commercial RNL: (a) 1^st^ heating curve; (b) cooling after melting; (c) 2^nd^ heating curve after cooling the melt, with the *T*_g_ marked by an inset expansion. |β| = 20 °C min^–1^.

**Figure 6:**
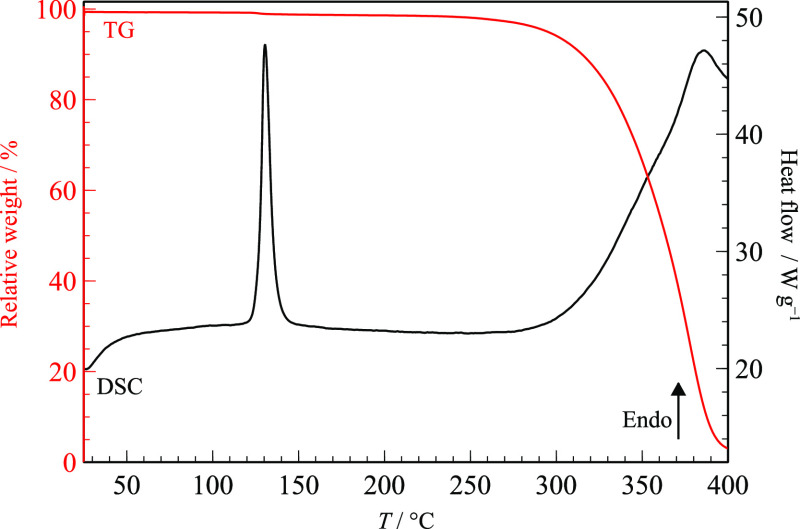
Thermogravimetric mass loss curve (red line) and DSC heating curve (black line) of commercial RNL. β = 20 °C min^–1^.

The DSC heating curve of RNL polymorph I, curve (a) in Figure 5, shows a single endothermic event corresponding to melting at *T*_fus_ = (118.5 ± 0.4) °C with Δ_fus_*H* = (54 ± 2) kJ mol^–1^. Thermogravimetric analysis, shown in Figure 6, did not reveal any weight loss before melting, as expected for a solvent-free form. No degradation was observed upon melting, permitting polymorph screening and amorphization methodologies involving the liquid. Decomposition is observed at *T* > 244 °C.

Different amorphization techniques were explored in this work, such as quench cooling and mechanochemistry.

#### 3.1.3 Quench Cooling

An amorphous solid phase was efficiently produced by cooling from the melt in sealed DSC pans at any of the scanning rates, showing a glass transition temperature at *T*_g_ = 20.5 °C when β = 20 °C min^–1^ (see Figure 5). Crystallization was not observed, neither while quench cooling, curve (b), nor after devitrification, curve (c).

#### 3.1.4 Cryo-Milling

**Figure 7:**
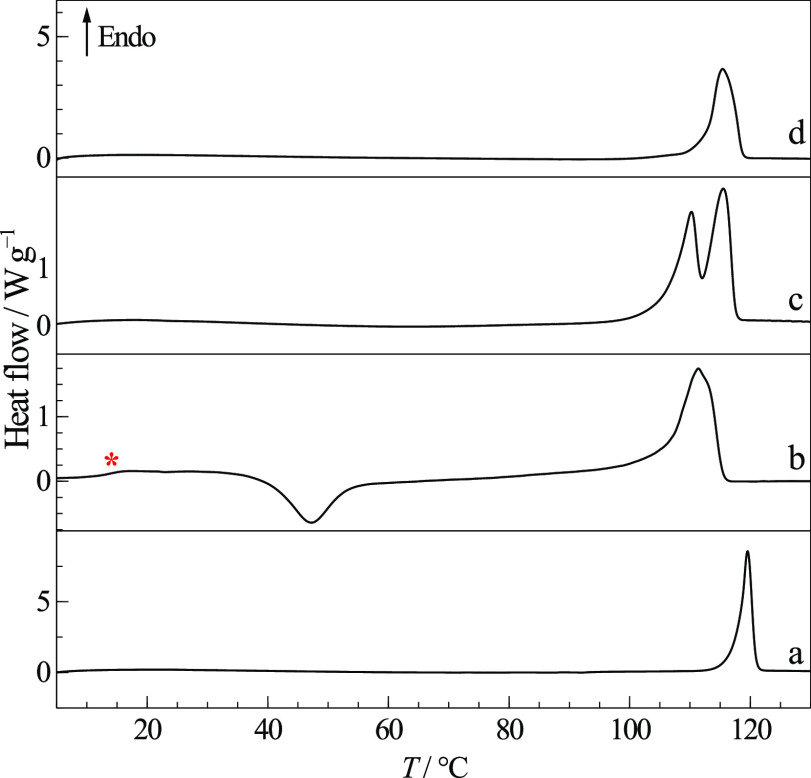
DSC curves of cryo-milled RNL: (a) commercial starting material, form I, for reference; (b) one cryo-milled sample (partially amorphous), with the *T*_g_ marked by a red asterisk (*); (c) another cryo-milled sample (mixture of forms II and III); (d) cryo-milled sample (c) kept at room temperature for a week. β = 20 °C min^–1^.

Amorphous RNL was also produced by cryo-milling, but removing the sample from the jars was challenging because the glass transition temperature of pure RNL is lower than room temperature. The amorphous phase was seldom recovered since, in most experiments, partial crystallization as form I took place before analysis. Nevertheless, it was possible to acquire experimental evidence of amorphization by cryo-milling in some experiments, either by FTIR-ATR, DSC, or XRPD. Spectrum (b) in Figure 4 presents the band broadening typical of disordered phases, compatible with the supercooled liquid. Some differences relative to spectrum (a), of RNL form I, can be noticed, like bathochromic shifts of the NH stretching from 3328 to 3288 cm^–1^ and of C=O stretching from 1683 to 1670 cm^–1^. Additionally, new spectral features are observed at 1501 and 1012 cm^–1^. These changes indicate different intermolecular interactions among RNL molecules. The glass transition of amorphous RNL is observed at 13 °C in the DSC thermogram (b) in Figure 7, a lower temperature than observed from quench cooling, showing that *T*_g_ depends on the amorphization method. Crystallization of the supercooled liquid is observed in the same curve, followed by a complex melting event at *T*_fus_ = 105 °C, which suggests the existence of other RNL polymorphs, unknown until now. VT-XRPD experiments starting from a cryo-milled RNL sample show a partially amorphous material that increases crystallinity when subject to heating from 20 to 100 °C, see Figure S2.

#### 3.1.5 Polymorphism after Cryo-Milling

The DSC heating curve of the material collected from another milling experiment, Figure 7c, shows two melting events with onsets at *T* = 105 and *T* = 113 °C, compatible with fusion of two polymorphic forms (III and II), different from form I (*T*_fus_ = 118 °C). XRPD confirmed the presence of new reflections (Figure 3c), although those assigned to form I (Figure 3b) are also present. The FTIR spectrum also confirmed the presence of new polymorphs (Figure 4c) by the appearance of new spectral features and some shifts relative to the characteristic bands of the initial form of RNL (Figure 4a). It should be remarked that these new spectral features and band shifts are closer to the disordered phases, but sharper, as expected for crystals, namely, at 1663, 1012, and 490 cm^–1^. Some broadening of spectral features remaining in Figure 4c is an indication of the residual amorphous content.

The metastable nature of forms II and III at room temperature was confirmed by XRPD, FTIR, and DSC results obtained in several experiments. It was observed that the new reflections in the XRPD diffractogram (Figure 3c), assigned to new forms II and III, decreased after storing the sample at room temperature for a week, while the characteristic peaks of form I increased (Figure 3d). The relaxation processes are evidenced in the calculated difference (d) – (c) in Figure 3e that separates the increasing signal of the stable form I from the decreasing metastable forms II and III, highlighting their characteristic reflections as downward peaks at (2θ = 10.6, 12.5, 13.5, 14.5, 15.5, 17.1, 17.5, 19.8, 20.1, 21.4, and 25.7°). Likewise, the FTIR-ATR spectrum (d) is very close to (a), as shown in Figure 4, also evidencing the relaxation of forms II and/or III obtained by cryo-milling into form I after a week. The room temperature metastability of form III relative to II was confirmed by DSC shown in Figure 7d. The single endothermic event observed at *T* = 113 °C corresponds to the fusion of form II, indicating prior evolution of III into II.

#### 3.1.6 Solid Forms from Solutions

Every mechanochemistry experiment produced amorphous phases. These frequently crystallized into form I or, occasionally, into a mixture of RNL polymorphs. Afterward, attempting to obtain new pure polymorphic or amorphous forms, screening was also performed by evaporation from different solvents (nonpolar, polar aprotic, and polar protic solvents) listed in Table S1. Recrystallization from most solvents leads to polymorph I. Particularly for dichloromethane, rapid evaporation at 60 °C under vacuum was attempted, producing a supercooled liquid that crystallized often as form I, and occasionally as form II, as shown in curve (c) in Figure S3. Unfortunately, the small yields and metastability of form II forbade X-ray diffraction experiments on these samples.

#### 3.1.7 Thermodynamic Stability Relationships

Burger and Ramberger’s thermodynamic rules provide a reliable way to ascribe stability relationships between polymorphs, either monotropic or enantiotropic.^28^ RNL forms I and II are monotropically related according to the heat of fusion rule since form I has higher temperature and higher heat of fusion (Table 2). Thus, form I is stable at all temperatures below the melting point, while form II is always metastable. The small difference of about 5 °C between *T*_fus_ of both polymorphs allows the estimation of the respective enthalpy of phase transition from the difference between the enthalpies of fusion: Δ_II⃗I_*H* = Δ_fus_*H*_II_ – Δ_fus_*H*_I_ = −27 kJ mol^–1^.

**Table 2:** Characteristic Fusion Parameters of RNL Polymorphs

	*T*_fus_/°C	Δ_fus_*H*/kJ mol^–1^
form I^a^	118.5 ± 0.4	54 ± 2
form II^b^	113.2 ± 0.5	27 ± 4

a *n* = 5.

b *n* = 3 (*n* = sample size).

### 3.2 l-Tryptophan

#### 3.2.1 Production of Amorphous Phases

Amorphization of TRP starting by quench cooling from the liquid is not feasible because it decomposes at melting temperature, as described in previous studies.^29,30^ Therefore, cryo-milling was elected to attempt amorphization. The starting material was identified as form α (CSD entry VIXQOK, CCDC 986568)^31^ and the method was successful, as confirmed by XRPD, DSC, and FTIR. Nevertheless, amorphous TRP showed some kinetical instability; occasionally, partial crystallization was observed, induced by sample handling.

#### 3.2.2 Thermal Behavior (VT-XRPD)

**Figure 8:**
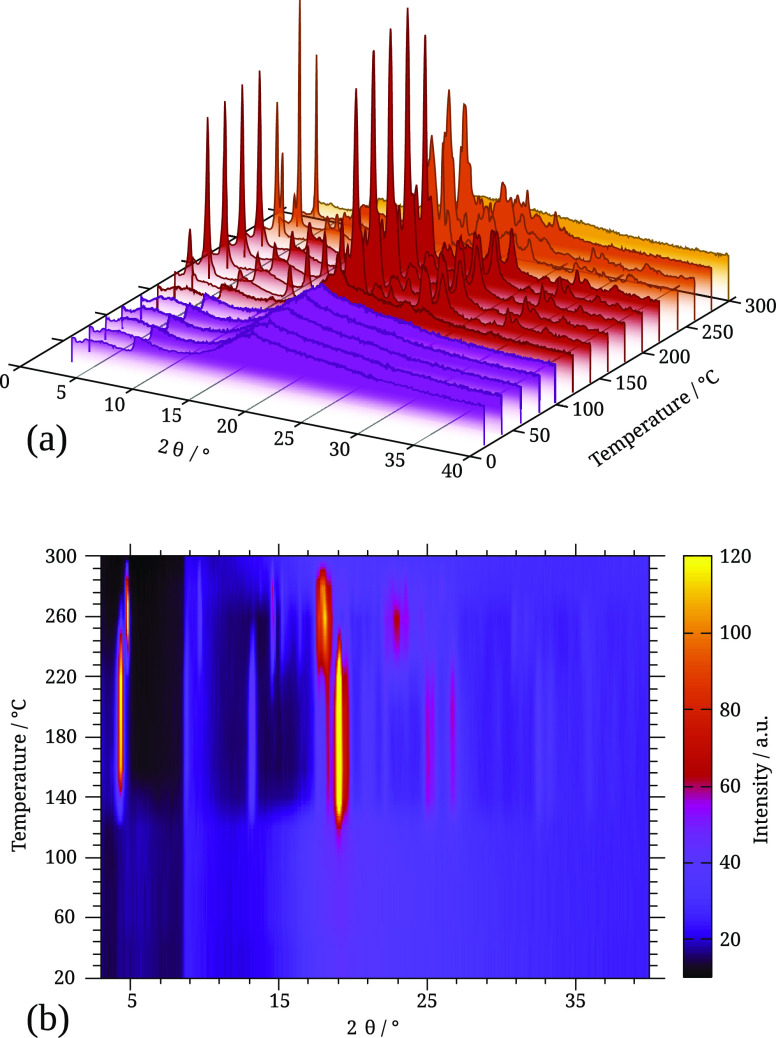
Stepwise relaxation of TRP solid forms analyzed by VT-XRPD. Heating from room temperature, amorphous TRP shows crystallization as polymorph β from 120 to 220 °C. This higher energy form then relaxes to form α, observed from 240 to 280 °C. At 300 °C, the sample is molten. (a) In the 3D plot, the amorphous form is observed in the purple lines, form α in red, form β in orange, and liquid in yellow. (b) Projection on the temperature-2θ plane, color-coded for intensity from dark purple to light yellow.

Cryo-milled amorphous samples were studied by VT-XRPD. Heating above 120 °C led to crystallization as polymorph β, previously reported by Al Rahal et al. (CSD entry VIXQOK02, CCDC 1937607).^20^ The experiment was stopped at 150 °C and the sample was kept at room temperature. After 5 days, it was verified that its diffractogram remained unaltered. As can be seen in Figure 8, further heating induced progressive relaxation to form α, at about 240 °C, and melting of this polymorph was observed below 300 °C.

#### 3.2.3 Thermal Behavior in Perforated DSC Pans

**Figure 9:**
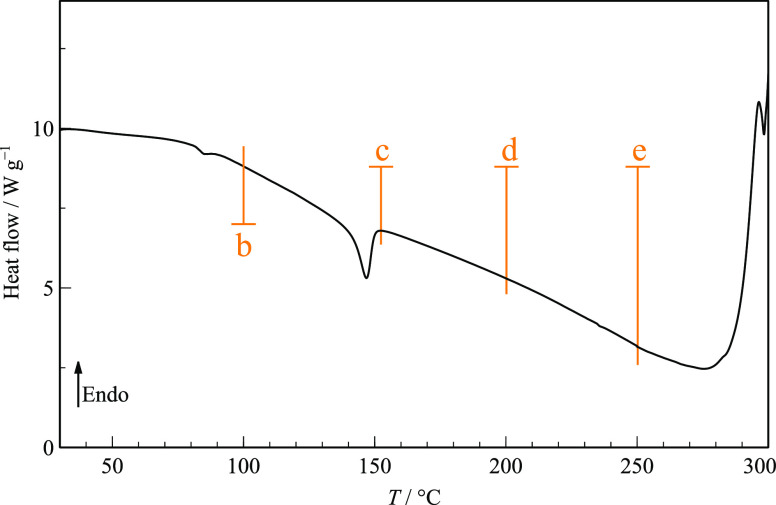
Typical DSC heating curve of cryo-milled TRP in perforated pans. Heating runs were stopped at temperatures indicated at points (b) to (e), the pans were opened to record their corresponding FTIR-ATR spectra, shown below in Figure 10. The final melting event before 300 °C was interrupted before completion to prevent sample decomposition and pan bursting inside the oven. β = 20 °C min^–1^.

**Figure 10:**
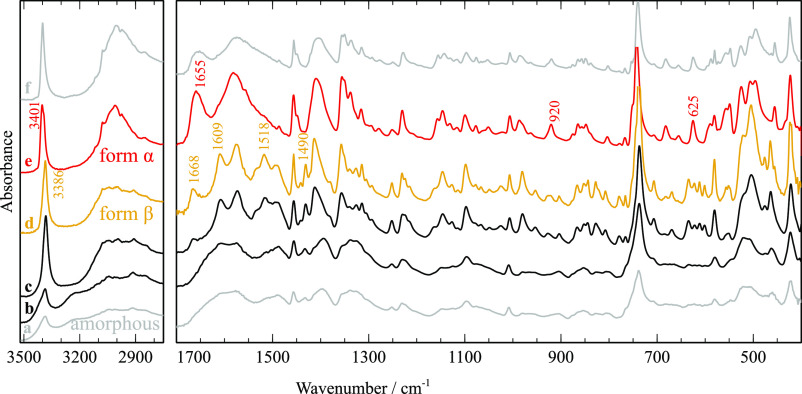
FTIR-ATR spectra of cryo-milled TRP taken from perforated DSC pans heated to different temperatures, marked in Figure 9: (b) 100 °C; (c) 150 °C; (d) 200 °C; (e) 250 °C. Spectra of amorphous (a) and crystalline (form α) TRP (f) shown for reference. Numeric labels mark frequencies characteristic of each polymorph α (red) or β (yellow).

This behavior was often reproduced in DSC experiments using perforated pans, although sample handling induced crystallization before some heating runs started, and no other events were observed except the melting of form α before 300 °C. Figure 9 shows a typical thermogram when starting from amorphous cryo-milled TRP. This is characterized by a crystallization event at about 150 °C and concur with previously published results under similar conditions.^32^ The changing nature of the solid phases was analyzed by FTIR-ATR spectra (Figure 10) taken after heating to different temperatures and opening the DSC pans.

The FTIR-ATR spectrum of the starting material, shown in Figure 10f, agrees with previous studies reported for zwitterionic crystalline form α dispersed in KBr.^33−36^ The successful amorphization of TRP by cryo-milling was confirmed by the typical broadening of spectral features seen in spectrum (a) in Figure 10. Moreover, some different band profiles suggest different short-range molecular interactions in the amorphous phase, compared to the initial form α.

Upon heating till 100 °C, band sharpening is observed in (b), caused by partial crystallization. The sharper features in spectra (c) and (d) provide evidence for complete crystallization, indicated by the exothermic event before 150 °C. Spectrum (d), taken after heating to 200 °C, matches the infrared spectrum of form β obtained by Liu and Li after sublimation.^37^ The similarity between the spectra of the amorphous material and of polymorph β, rather than α, indicates short-range molecular order in the glass closer to the higher-energy crystalline form β. Further changes observed in the FTIR-ATR spectrum (e), obtained after heating till 250 °C, reveal progressive relaxation from polymorph β to α, not visible in the DSC thermogram.

Summarizing, all experimental techniques evidence the crystallization of the amorphous solid to a metastable polymorph β below 150 °C, followed by further relaxation to the more stable form α.

#### 3.2.4 Thermal Behavior in Sealed DSC Pans

When sealed pans were used, the observed thermal behaviors were less predictable. Several examples are provided as Supporting Information in Figures S4 and S5. Crystallization occurs frequently before heating started, and no other events except the melting of form α were observed, as shown in curve (a) in Figure S4. Occasionally, a sharp exothermic peak appeared at temperatures below 60 °C. Uncorrelated with the latter, an endothermic feature can be observed either around 100 or 150 °C. These events were more frequent in pans of 10 rather than 50 μL capacity and independent of the heating rate; see Figure S5. Pan hermeticity, on the other hand, seems to be a decisive factor in amorphous relaxation kinetics.

#### 3.2.5 Unexpected Appearance of the Neutral Form of TRP in a Solid Phase

**Figure 11:**
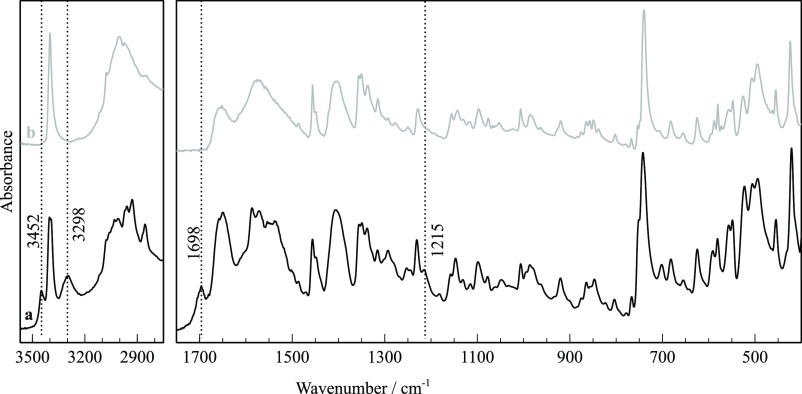
(a) FTIR-ATR spectrum of cryo-milled TRP taken from a sealed DSC pan heated to 160 °C highlighting additional features ascribed to a small fraction of neutral TRP dimers in the deposited solid phase after sublimation. (b) Spectrum of TRP form α shown for comparison.

All FTIR-ATR spectra taken after opening the sealed pans throughout various stages during the heating ramps confirmed the presence of form α. Whimsically, a different spectrum, presented in Figure 11, was obtained unoften, revealing additional signals besides those assigned to that same polymorph. The extra bands are neither compatible with spectra of form β, nor the amorphous solid, nor decomposition products assigned in previously reported TG-FTIR experiments.^38^ The origin of those four bands is compatible with the extraordinary prevalence of neutral, unionized TRP in a condensed phase. While amino acids are generally considered exclusively zwitterionic as pure solids, a few have been detected by infrared spectroscopy in neutral forms under special conditions, namely, after deposition from sublimated vapors onto cryogenic substrates. Such was the case of glycine, dimethylglycine, sarcosine, and alanine.^39−41^ The most prominent bands assigned in those studies are in complete agreement with the additional spectral features we observed in our spectrum of TRP. In the high-frequency region, the strong and sharp indole NH stretching band at 3401 cm^–1^ is flanked by two new bands that must be assigned to neutral amino acid groups, non-H-bonded OH (3452 cm^–1^), and NH_2_ (3298 cm^–1^), matching the same profile observed in spectra of TRP in cryogenic Ar and Xe solid matrices.^42^ Additionally, another new band appears at 1698 cm^–1^ that must be therefore assigned to C=O stretching of the neutral molecule, at a much higher frequency than the carboxylate ion asymmetric stretching at 1655 cm^–1^ of the zwitterion. The other extra band at 1215 cm^–1^ can be assigned to the ν(C–O) + δ(COH) combination mode, as suggested by DFT calculations of infrared spectra for the neutral amino acid dimers.^40^

Taking into account the much greater stability of the zwitterionic form of TRP in solid phases, some explanation should be provided for its occurrence mixed with small quantities of the neutral form in DSC experimental conditions. The different thermal behavior observed in sealed DSC pans supports that there is pressure build-up inside caused by some sublimation. One can assume that the higher partial pressure of TRP vapors in this case leads to the formation of dimers of neutral TRP that are subsequently deposited as the solid material. Zwitterions are formed after deposition by proton transfer from the COOH to the NH_2_ groups of adjacent molecules in the solid phase,^40^ which can be hindered if neutral TRP molecules are deposited from the gas phase as centrosymmetric dimers. This mechanism is supported by the better agreement of the DFT calculations of infrared spectra of neutral amino acid dimers with the experimental spectral features observed in solid phases deposited on cold substrates.^40^ The reduced molecular mobility caused by the larger TRP substituent (indole) when compared to alanine (CH_3_) and glycine (H) explains why its neutral form survives at higher temperature, corroborating previous analogous findings for the smaller amino acids.^39^

### 3.3 Co-Amorphous Systems of RNL-TRP

#### 3.3.1 Production of Co-Amorphous Systems with Different Molar Ratios

Co-amorphous systems of ranolazine and tryptophan were also investigated to increase the kinetical stability of the disordered solid phase, as well as to improve RNL low aqueous solubility.

**Figure 12:**
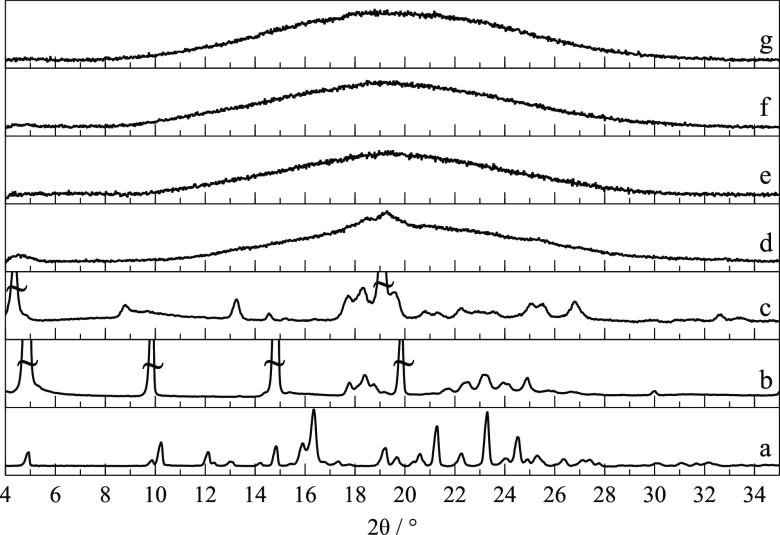
X-ray powder diffractograms of (a) RNL polymorph I; (b) TRP polymorph α; (c) TRP polymorph β; (d) cryo-milled RNL-TRP 1:3; (e) cryo-milled RNL-TRP 1:2; (f) cryo-milled RNL-TRP 1:1; and (g) cryo-milled RNL-TRP 2:1.

Figure 12 shows the XRPD patterns of four cryo-milled RNL-TRP binary mixtures, in different molar ratios. Complete amorphization was achieved for the 2:1, 1:1, and 1:2 molar ratios, whereas residual reflections attributed to TRP polymorph β in the diffractogram of the RNL-TRP 1:3 mixture indicate partial crystallinity. Hence, this mixture was excluded from further investigation.

**Figure 13:**
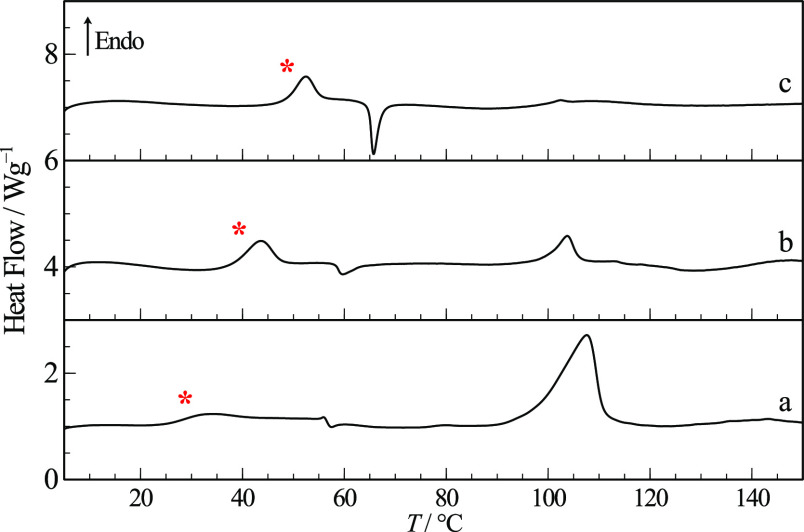
DSC heating thermograms of co-amorphous RNL-TRP in different molar ratios: (a) 2:1; (b) 1:1; (c) 1:2. The glass transitions are marked with asterisks (*). β = 10 °C min^–1^.

The DSC thermograms in Figure 13 show that the binary amorphous mixtures (2:1, 1:1, and 1:2) have a single glass transition event, as expected for a co-amorphous system, with *T*_g_ values increasing with the TRP content. These values are higher than the glass transition temperature of pure amorphous RNL obtained by cryo-milling (*T*_g_ = 13 °C) and, consequently, higher kinetic stability is expected.

#### 3.3.2 Dependence of *T*_g_ with Composition

**Figure 14:**
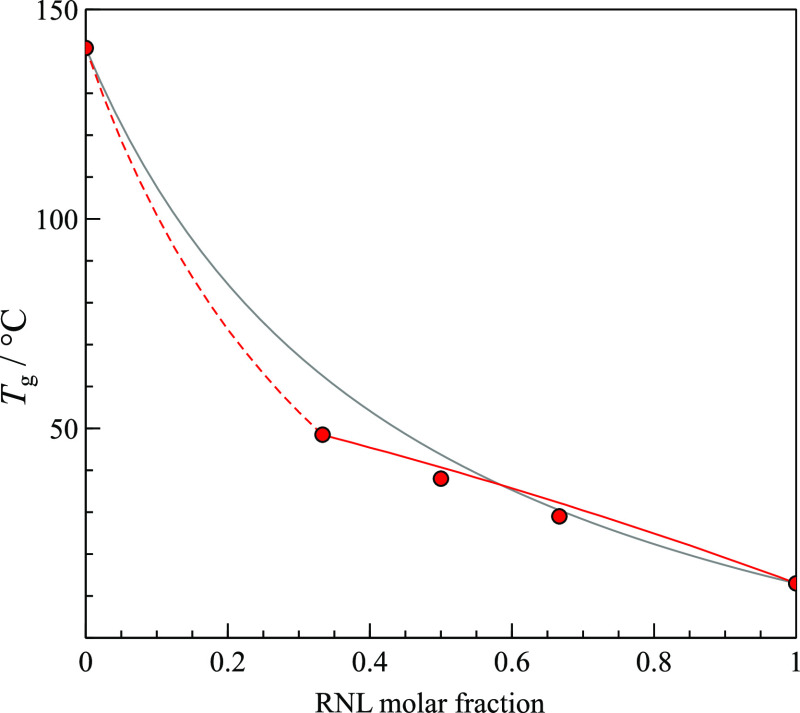
Experimental *T*_g_ values (red circles) show negative deviation from the Gordon–Taylor equation (gray line) but follow the modified Gordon–Taylor approach (red lines). No experimental *T*_g_ values were determined for mixtures with RNL molar fraction below 1/3 because they crystallize before reaching those temperatures (dashed red line).

The experimental *T*_g_ values were compared with those predicted by the Gordon–Taylor equation (eq 1):^43^
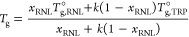
1where *x*_RNL_ is the RNL weight fraction in the mixture, the component with lower glass transition temperature, and ° denotes properties of pure components. Assuming validity of the Simha–Boyer rule, the value of the *k* constant can be estimated by eq 2:^43^
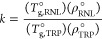
2where ρ_RNL_° and ρ_TRP_° are the densities of the two pure amorphous phases, assumed as approximately the same as those of the respective crystalline phases: TRP polymorph α^31^ and RNL solved in this work. Since the glass transition of TRP was not observed in this work, *T*_g,TRP_° = 140.8 °C was taken from the literature.^32^ Experimental *T*_g_ values presented in Table 3 show deviation from the ideal behavior, progressively negative as the TRP ratio increases. This prompted the use of the modified Gordon–Taylor approach where the amorphous mixture with the optimal molar ratio and the excess component are considered as pure substances.^44^ The 1:2 RNL–TRP mixture was selected for this purpose because of its greater stability over time (see below) and its higher *T*_g_, also most deviated from the predicted value. Following this approach, the *T*_g_ of the other mixtures then nicely fit the Gordon–Taylor equation, as also can be seen in Figure 14.

**Table 3:** Experimental *T*_g_ of RNL-TRP Co-Amorphous Systems and Pure Components and Deviations from Gordon–Taylor (Δ_G-T_*T*_g_) and Modified Gordon–Taylor (Δ_mod.G-T_*T*_g_) Behavior

RNL-TRP	*T*_g_/°C	Δ_G-T_*T*_g_/°C	Δ_mod.G-T_*T*_g_/°C
pure TRP	140.8^a^		
1:2	48.5 ± 1.4	–14.0	
1:1	38.0 ± 1.2	–5.6	–2.7
2:1	28.9 ± 0.8	–1.6	–3.3
pure RNL	13.0 ± 0.2		

a Taken from ref (32). The modified Gordon–Taylor approach considers the 1:2 RNL-TRP co-amorphous system and the excess RNL or TRP as pure components.

Negative deviation from the ideal Gordon–Taylor behavior can be generally explained by weaker intermolecular interactions in the RNL-TRP mixture than in the pure components, resulting in endothermic enthalpy of mixture of the amorphous components. Nevertheless, the glass transition being essentially an entropy-driven process, the deviation from the idealized behavior may result from entropy effects beyond the combinatorial mixture, namely, when some exo or endothermic mixing occurs. While stabilizing (exothermic) interactions in the glass mixture result in negative entropy of mixing, destabilizing (endothermic) interactions lead to positive configurational entropy of mixing available to the supercooled liquid that tends to shift *T*_g_ to a lower value than expected by the simple idealized case of an athermal solid mixture.^45^

The spontaneous crystallization observed for lower RNL molar ratios (dashed line in Figure 14) can be induced by the very different molecular sizes of the components. As the amorphous mixture becomes richer in TRP, their smaller molecules are not segregated as effectively by the larger RNL units and thus tend to aggregate and crystallize as form β, as was referred above and noticed in Figure 12d.

#### 3.3.3 Physicochemical Characterization of RNL-TRP Co-Amorphous Systems

**Figure 15:**
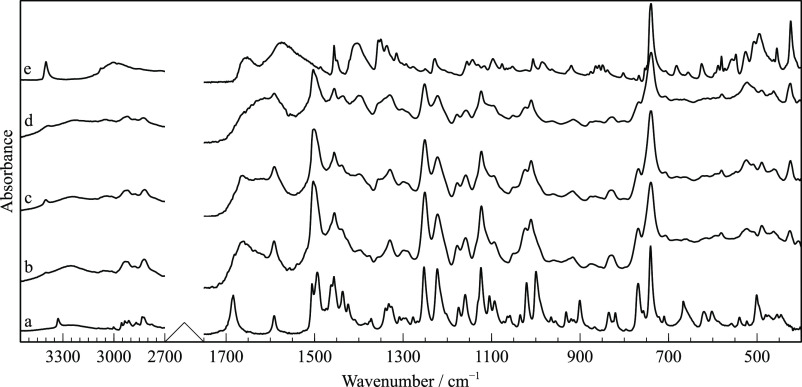
FTIR-ATR spectra of pure compounds and co-amorphous mixtures: (a) commercial RNL; (b) RNL-TRP (2:1); (c) RNL-TRP (1:1); (d) RNL-TRP (1:2); (e) commercial TRP (form α).

The FTIR-ATR spectra of RNL-TRP mixtures presented in Figure 15 show a typical band broadening characteristic of amorphous solids and a significant shift of the RNL C=O stretching band to lower frequencies. These changes can be attributed to amorphization and to intermolecular interactions with TRP. Other distinguishing spectral features are caused by different molecular arrangements in the co-amorphous mixtures when compared to the crystalline starting materials.

**Figure 16:**
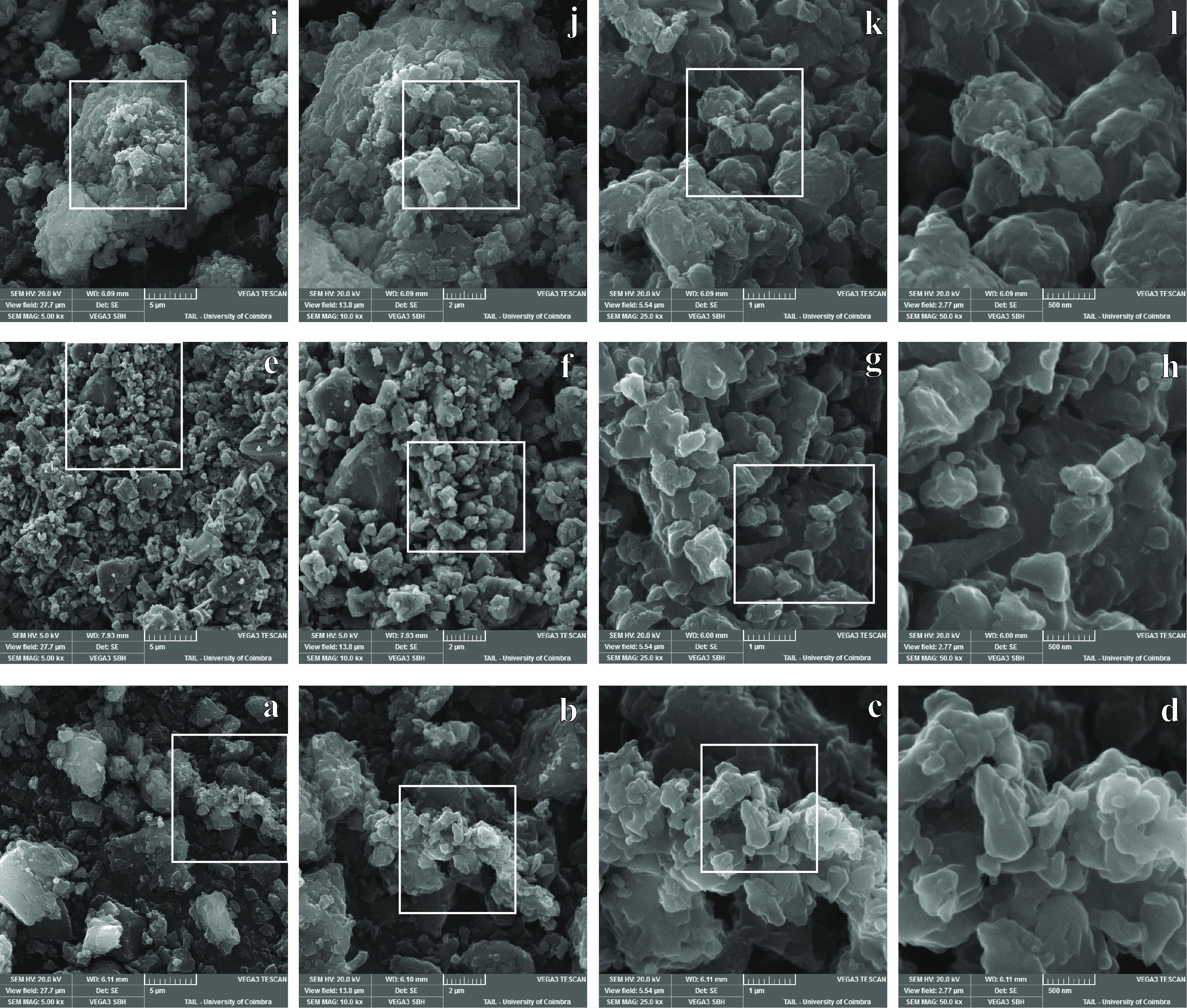
SEM images of RNL CM: (a) 5000×, (b) 10,000×, (c) 25,000×, and (d) 50,000× of RNL-TRP 1:2: (e) 5000×, (f) 10,000×, (g) 25,000×, (h) 50,000×; TRP CM: (i) 5000×, (j) 10,000×, (k) 25,000×, (l) 50,000×. A white square indicates the area amplified in the rightward frame.

Particle sizes and morphology were analyzed by SEM, as shown in Figure 16. The SEM images of cryo-milled samples revealed particle sizes from 5 to 0.5 μm. As can be seen in frames (e) and (f), the particle division was achieved more evenly for the RNL-TRP mixture, leaving very few larger than 2 μm, than for the pure components. Particle size reduction and the wrinkled and irregular surfaces of cryo-milled RNL-TRP 1:2 may contribute to the increase in the surface area, leading to the enhancement of solubility.

#### 3.3.4 Stability of RNL-TRP Co-Amorphous Systems

**Figure 17:**
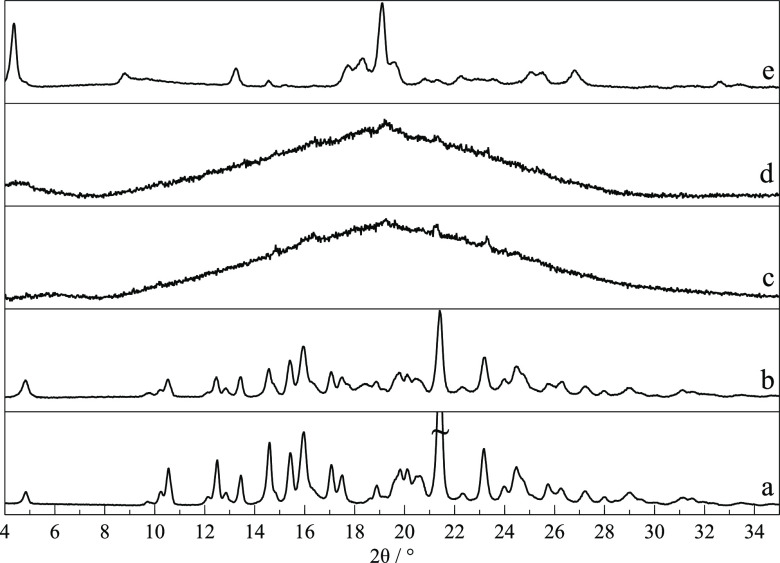
X-ray powder diffractograms of RNL-TRP samples, in different molar ratios, after 2 months kept at room temperature (b), (c), and (d), compared with crystalline samples of pure compounds, (a) and (e): (a) mixture of RNL polymorphs obtained from amorphous relaxation (see above); (b) RNL-TRP CM 2:1; (c) RNL-TRP CM 1:1; (d) RNL-TRP CM 1:2, and (e) TRP polymorph β.

The kinetic stability of RNL-TRP co-amorphous samples was also evaluated, for samples kept in closed vials at ambient conditions. After two months, the 2:1 co-amorphous system crystallized in a mixture of different RNL polymorphs; see diffractogram (b) in Figure 17. Samples with the other two molar ratios of RNL-TRP investigated in this work remained essentially amorphous, despite the appearance of residual diffraction peaks characteristic of the TRP β polymorph.

#### 3.3.5 Screening Multicomponent Solid Forms under Different Conditions

Several experiments were also performed to screen other possible multicomponent solid-forms starting from the same materials. Different experimental methods were explored, such as neat and ethanol-assisted grinding (30 Hz, 60 min at room temperature) and slurry with different solvents (ethyl acetate, ethanol, acetonitrile) for 48 h. All these experiments produced physical mixtures of RAN form I and TRP form α.

### 3.4 Aqueous Solubility Studies

This work also aims to expand the RNL solid forms landscape that may improve its aqueous solubility. The 1:2 RNL-TRP co-amorphous system has the highest glass transition temperature, contributing to a higher kinetic stability of the amorphous phase when stored at room temperature. Therefore, this mixture was chosen to evaluate the possible RNL aqueous solubility enhancement.

Apparent aqueous solubilities of RNL as pure crystal and in co-amorphous mixture were determined at 25 °C by the shake-flask method. No changes were observed in the solid forms at equilibrium with the solutions by the end of the experiments; Figure S6. The solubility of crystalline RNL is (250 ± 20) μg mL^–1^, classified as sparingly soluble.^46^ Using the co-amorphous system RNL-TRP 1:2, a significant enhancement of apparent solubility is observed – RNL apparent solubility is increased to (750 ± 80) μg mL^–1^.

## 4 Conclusions

**Scheme 1:**
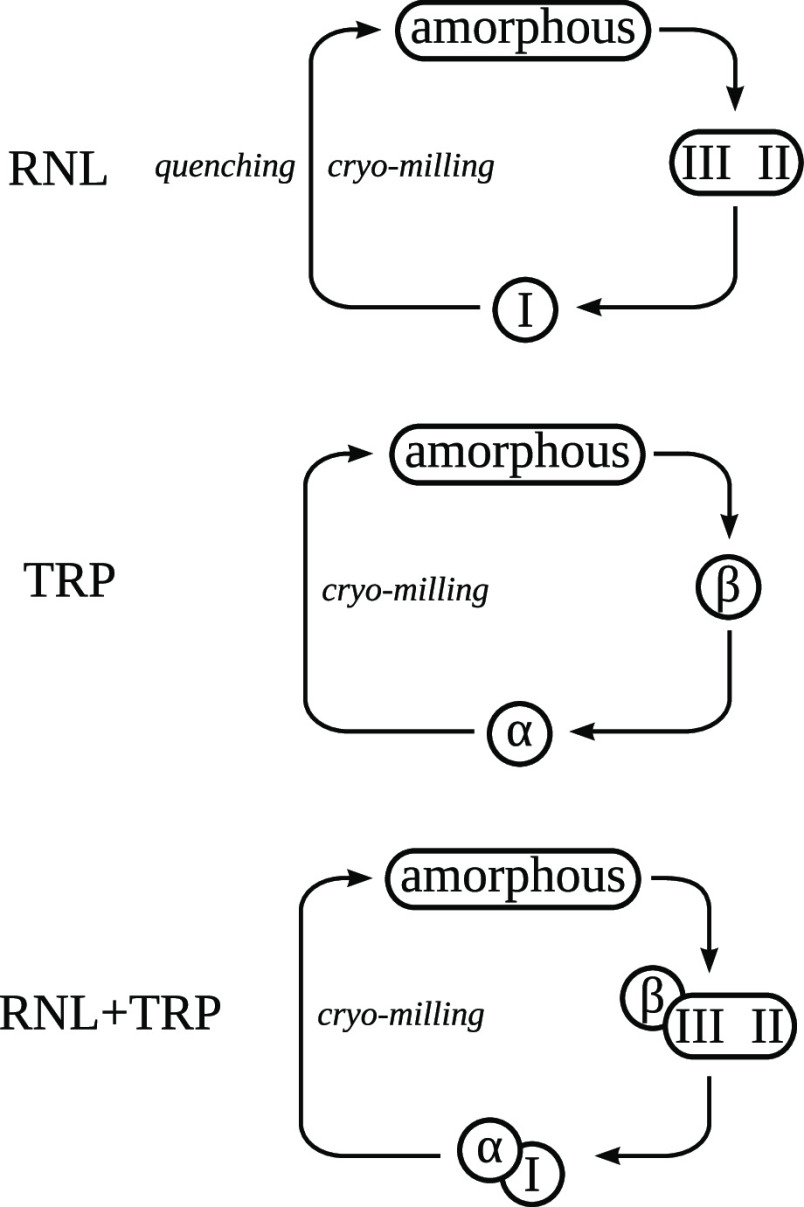
Summary of Processes Leading to Pure and Multicomponent Amorphous Forms of RNL and TRP and Their Relaxation to Metastable and Ultimately Low-Energy Forms

All amorphous phases produced in this work showed relaxation to crystals, sooner or later, depending on their kinetic stability: usually first to high-energy forms and then to the stable, low-energy polymorphs, as summarized in Scheme 1.

Amorphous RNL was successfully produced both by melt quenching and cryo-milling. The quench-cooled sample did not crystallize in subsequent heating runs until 130 °C. On the other hand, amorphous RNL produced by cryo-milling, *T*_g_ = 13 °C, relaxes to crystalline forms, either standing at ambient conditions or upon heating, which can be induced by the leftover residual crystalline nuclei in the milled samples. Moreover, this recrystallization process unraveled complex polymorphism: amorphous phase relaxation led to the discovery of the new metastable RNL polymorphs II and III, melting in close proximity, respectively, *T*_fus_ = 112 and *T*_fus_ = 105 °C. The high-energy RNL forms II and III further relax to the low-energy form I after a week. Additionally, in this work, the crystalline structure of a lower energy RNL polymorph, form I, *T*_fus_ = 118.5 °C, was solved by SCXRD for the first time.

TRP amorphous phases produced by cryo-milling showed some kinetical instability and partial crystallization was sometimes observed, induced by sample handling. Different experimental conditions (e.g., use of DSC perforated or sealed pans) gave rise to different amorphous relaxation paths. Under nonconfinement conditions, relaxation to the metastable polymorphic form β was observed, and subsequent transformation to polymorph α was documented for the first time. In most DSC experiments performed in sealed pans, crystallization occurs before heating started, and no other events except the melting of form α were observed. Occasionally, uncorrelated exothermic and endothermic features, below 60 °C and either around 100 or 150 °C, respectively, are observed that need further investigation. FTIR-ATR spectra were taken for different samples after opening the sealed pans throughout various stages during the heating runs. Serendipitously, a spectrum was obtained revealing, besides the signals assigned to polymorph α, extra bands that point out to the extraordinary prevalence of neutral, unionized TRP in the condensed phase.

TRP was chosen as a co-former with the intent to act as a stabilizing agent for the amorphous phase of RNL, producing co-amorphous systems with the highest possible *T*_g_, and inhibiting the relaxation to the crystalline state. Considering these goals, the RNL-TRP 1:2 co-amorphous system was selected as the most suitable for a possible pharmaceutical formulation, since it has the highest *T*_g_ value and the highest kinetic stability. Additionally, a considerable enhancement of RNL apparent aqueous solubility is achieved when it is included in this co-amorphous phase.

## Supporting Information Available

Calibration curves for solubility studies; VT-XRPD diffractograms of cryo-milled RNL; DSC heating curves of RNL; examples of DSC heating curves of cryo-milled TRP in sealed pans; β = 20 °C min^–1^; DSC heating curves of cryo-milled TRP in sealed pans at various scanning rates; XRPD of solids recovered after solubility studies; and RNL polymorph screening results from solvent evaporation (PDF)
