# Supplementary material for: Amorphous Solid Forms of Ranolazine and Tryptophan and Their Relaxation to Metastable Polymorphs
Source: Cryst Growth Des. 2023 Aug 18;23(9):6679–91. doi: 10.1021/acs.cgd.3c00565 (PMC10486308; doi:10.1021/acs.cgd.3c00565)
Supplement: Supplementary file 1 — cg3c00565_si_001.pdf [file cg3c00565_si_001.pdf]

Supporting Information

Amorphous solid forms of ranolazine and tryptophan, and their relaxation to metastable polymorphs

Joana F. C. Silva,[a] Pedro S. Pereira Silva,[b] Manuela Ramos Silva,[b]
Elvira Fantechi,[c] Laura Chelazzi,[c] Samuele Ciattini,[c]
M. Ermelinda S. Eusébio,[a]* Mário T. S. Rosado[a]*

[a]CQC-IMS, Departamento de Química, Faculdade de Ciências e Tecnologia, Universidade de Coimbra, Rua Larga, 3004-535 Coimbra, Portugal.
[b]CFisUC, Departamento de Física, Faculdade de Ciências e Tecnologia, Universidade de Coimbra, Rua Larga, 3000-370 Coimbra, Portugal.
[c]Centro di Cristallografia Strutturale (CRIST), Università degli Studi di Firenze, Via della Lastruccia 3, 50019, Sesto Fiorentino, Firenze, Italy.

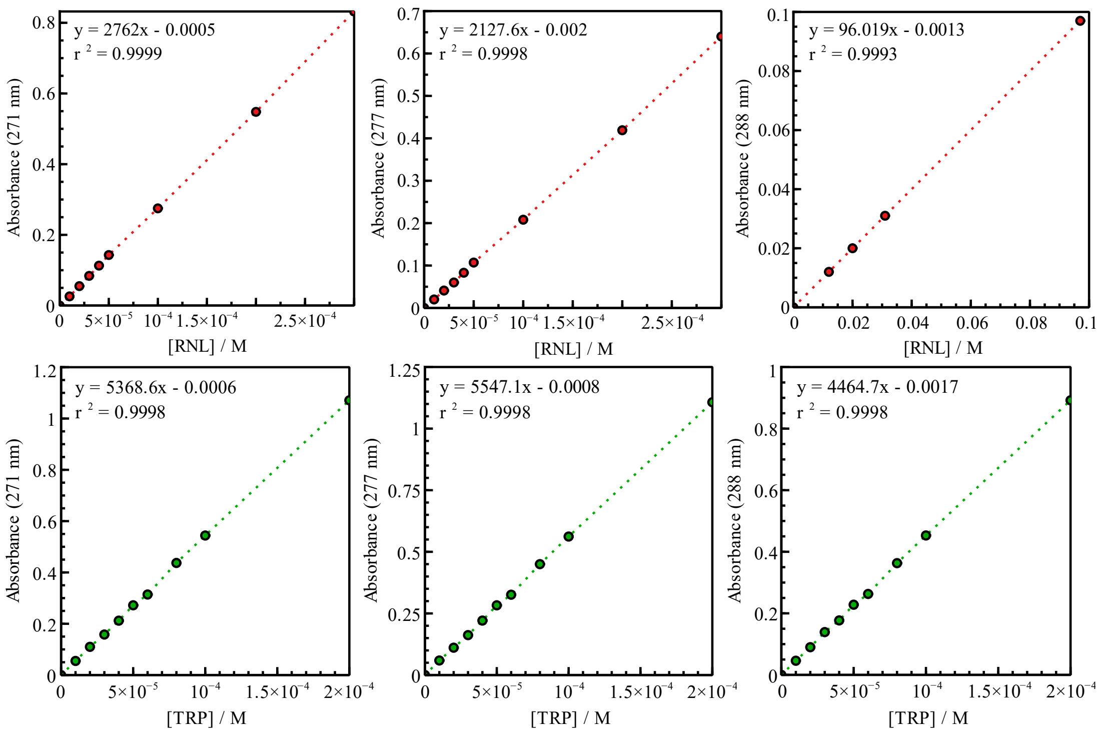

Figure S1. Calibration curves (absorbance vs. molar concentration) for RNL and TRP quantification in solubility studies.

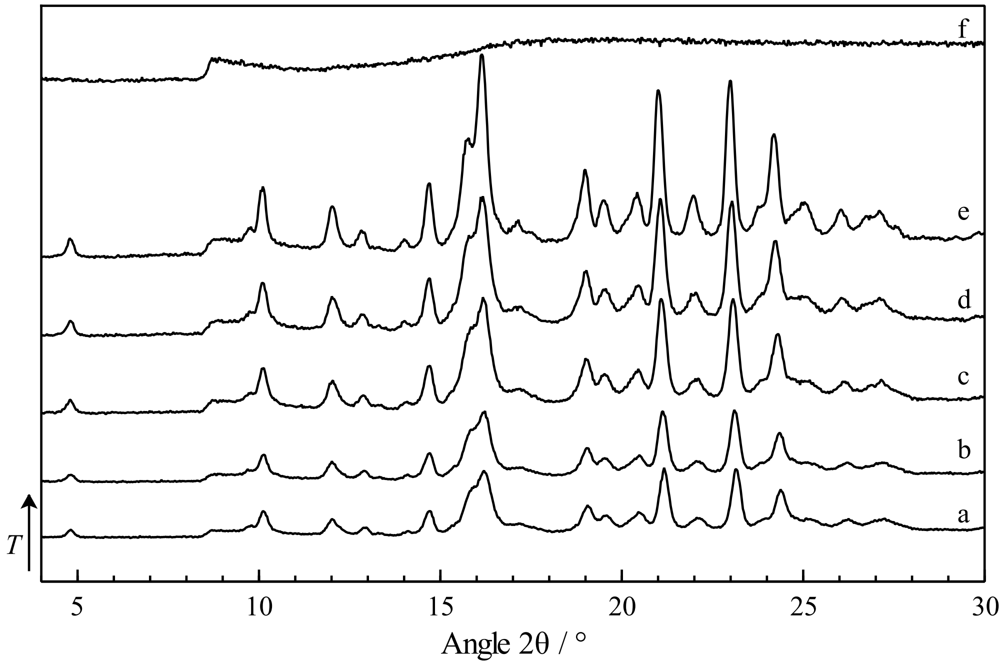

Figure S2. VT-XRPD diffractograms of cryo-milled RNL collected from room temperature to 120 °C: a) 20 °C, b) 40 °C, c) 60 °C, d) 80 °C, e) 100 °C, and f) 120 °C.

Table S1. RNL polymorph screening results from slow evaporation from solutions of different solvents.

**Solvent**	**Molecular formula**	**Boiling point / °C**	**RNL polymorph**
Non-polar solvents			
toluene	$C_6H_5CH_3$	111	**I**
Polar aprotic solvents			
dichloromethane	CH_2Cl_2	40	**II**
tetrahydrofuran	C_4H_8O	66	**I**
acetonitrile	CH_3CN	82	**I**
Polar protic solvents			
ethanol	CH_2CH_2OH	79	**I**
methanol	CH_3OH	65	**I**

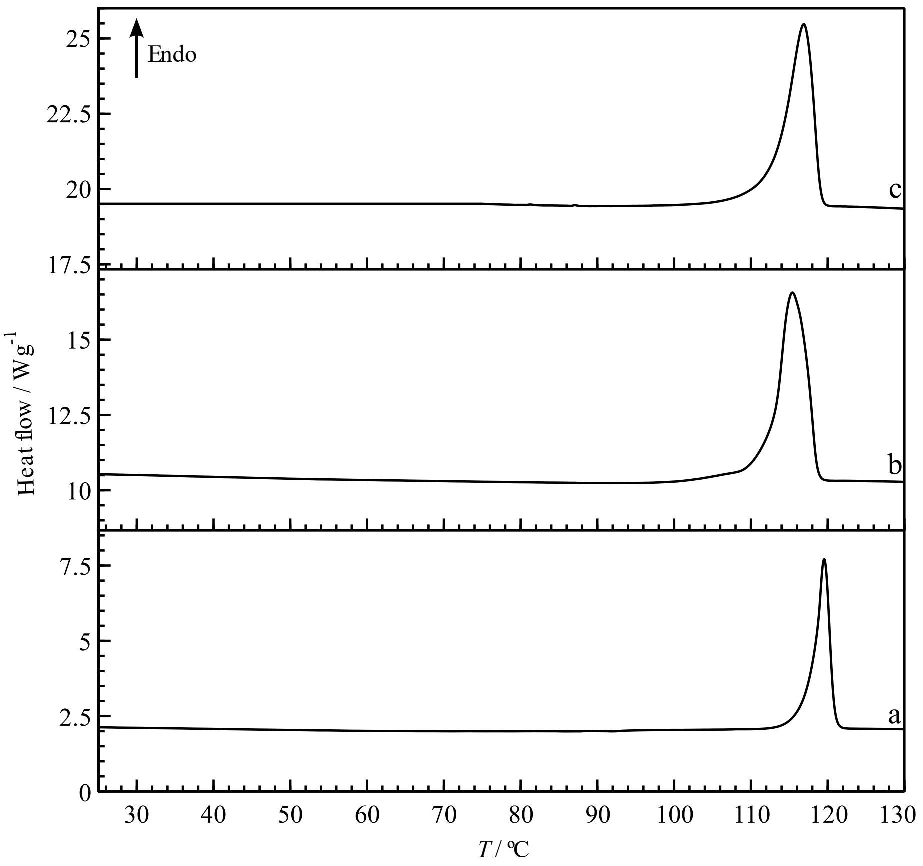

Figure S3. DSC heating curves of RNL a) commercial sample; b) after 1 month relaxation of a cryo-milled sample; c) obtained by rapid evaporation of a dichloromethane solution under vacuum at T = 60 °C. $\beta = 20$ °C min^{-1}.

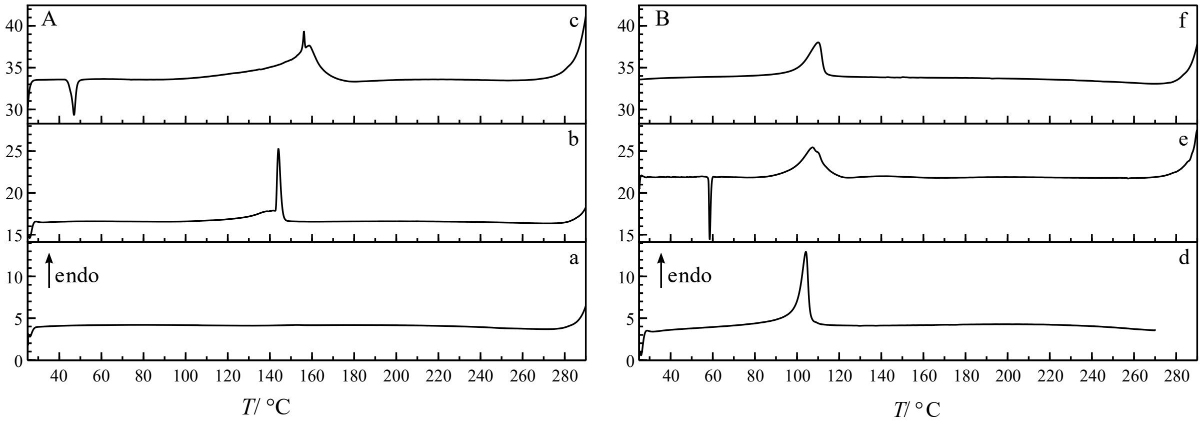

Figure S4. Several examples of DSC heating curves of cryo-milled TRP in sealed pans. Curve (a) shows the most frequently observed behavior (crystallization in TRP form α before heating started). The final melting event before 300 °C was interrupted before completion to prevent sample decomposition and pan bursting inside the oven. $\beta = 20$ °C min^{-1}.

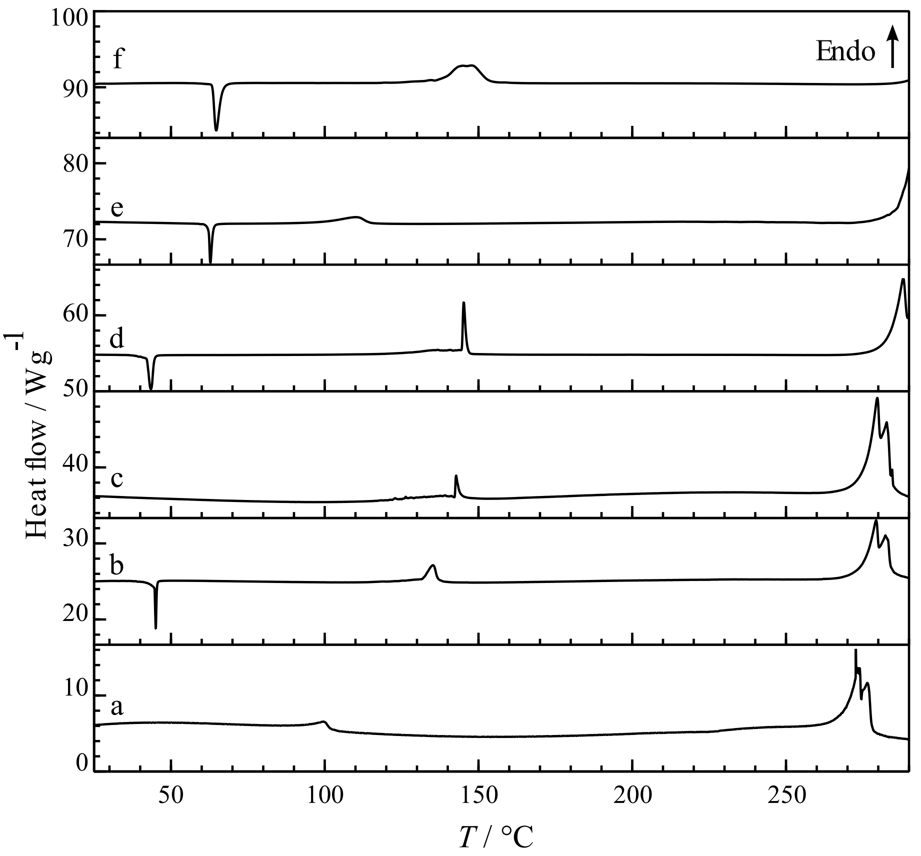

Figure S5. DSC heating curves of cryo-milled TRP, in sealed pans, showing that the observed thermal behaviors are independent of the heating rate. Heating rates investigated: a) 2; b) 5; c) 5; d) 10; e) 15 and f) $\beta = 30$ °C min^{-1}. Whenever possible, the final melting event was interrupted to prevent sample decomposition and pan bursting.

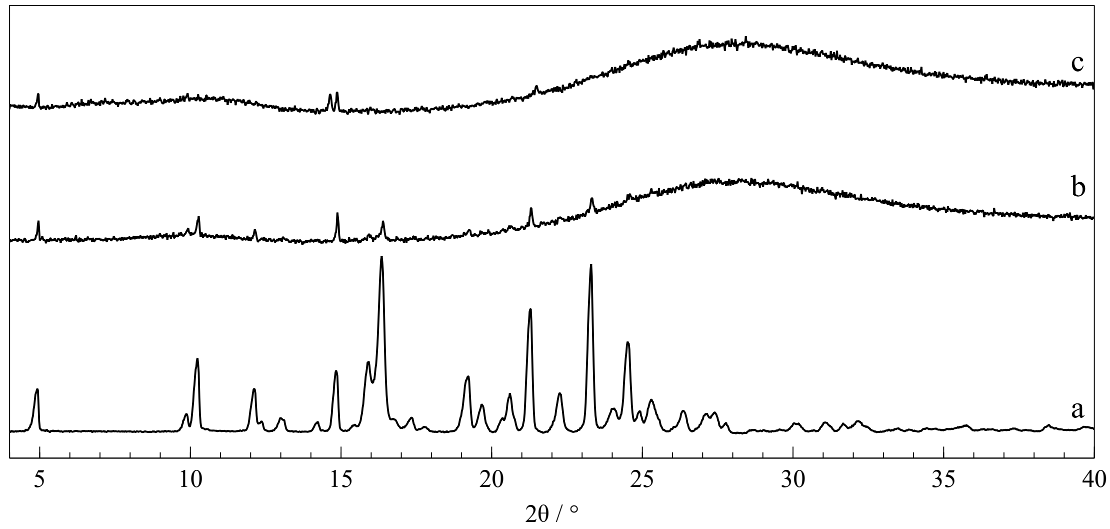

Figure S6. XRPD of commercial ranolazine (a) and solids recovered after solubility studies of RNL (b) and of 1:2 RAN-TRP co-amorphous system (c).
